# Determination of the Composite Panel Moulding Pressure Value

**DOI:** 10.3390/polym14122392

**Published:** 2022-06-13

**Authors:** Andrii Kondratiev, Václav Píštěk, Oleksii Vambol, Yurii Otrosh, Pavel Kučera, Ondřej Kučera

**Affiliations:** 1 Department of Building Technology and Construction Materials, O.M. Beketov National University of Urban Economy in Kharkiv, Marshal Bazhanov Str. 17, 61002 Kharkiv, Ukraine; andrii.kondratiev@kname.edu.ua (A.K.); olexii.vambol@khai.edu (O.V.); 2 Institute of Automotive Engineering, Brno University of Technology, Technická 2896/2, 616 69 Brno, Czech Republic; pistek.v@fme.vutbr.cz (V.P.); kucera@fme.vutbr.cz (P.K.); 3 Department of Composite Structures and Aviation Materials, National Aerospace University “Kharkiv Aviation Institute”, Chkalova Str. 17, 61070 Kharkiv, Ukraine; 4 Department of Fire Prevention in Settlements, National University of Civil Defence of Ukraine, Chernishevska Str. 94, 61023 Kharkiv, Ukraine; yuriyotrosh@gmail.com

**Keywords:** process parameters, equipment, thermoelasticity, temperature differential

## Abstract

Currently, prefabricated panel structures are typical products made of polymeric composite materials. The integrity of the composite panels, their structure and accuracy of making a contour are largely associated with the manifestation of residual technological stresses. The above phenomena and associated stress-strain behaviour inevitably occur in the process of moulding of the composite products. However, their value, nature, time of occurrence and dynamics of growth can be fully controlled and regulated. The paper deals with the study of the effect of moulding pressure on the quality of a composite product. A dependence is presented that allows us to determine the time for the degassing of the polymeric composite material package at the given temperature and pressure to obtain a monolithic and nonporous structure. It is shown that the peak of the maximum volatile-matter yield for the considered binder types lies in the temperature range where the degree of curing does not exceed 10%; that is, the viscosity values do not prevent the removal of volatile fractions. The effect of moulding pressure on the values of the volume content of the reinforcing material has been studied, and the dependence of the required thickness of the absorbent layer on the parameters of the package of polymer composite material and pressure has been obtained. The dependence of the required thickness of absorbent layer on the parameters of the package of polymeric composite material and pressure has been obtained. The mathematical model developed by us provides an opportunity to predict the stress-strain behaviour of a composite structure at any time during the moulding process. The model is closely related to chemo-viscous and thermal models. It allowed us to synthetize a method for choosing the rational parameters of the moulding process (temperature, pressure, and time), materials of additional layers and equipment. The experiments proved the presence of several defects, such as de-lamination of edges, waviness, swelling and poor adhesion of upper layers in the specimen of the composite panel cooled stepwise in the absence of the vacuum pressure. The surface quality of the specimen of the panel cooled stepwise under vacuum pressure was significantly better, and no visible defects were observed. The obtained theoretical values of deflections, considering the change in physic-mechanical characteristics that depend on the temperature and rheonomic properties of the material, showed an error that did not exceed 7%, compared to the experimental data. Our results can be applied at the enterprises engaged in designing and manufacturing panel structures of polymeric composite materials.

## 1. Introduction

Polymeric composite materials (PCM), owing to their unique properties, are widely used in many industries [1,2]. PCM are increasingly used in transport and power engineering, construction, electronics, and manufacturing of pipes and tanks [3,4,5]. By now, further improvement and expansion of the scope of application of these materials is one of the most pressing problems [6,7]. An important role in the PCM products’ payback is played by the energy and labour costs [8,9]. The process of moulding of a part after which it receives a given shape and properties takes a lot of time and energy [10,11]. Currently, most enterprises use energy-intensive and time-consuming temperature-time regimes [12,13], and sometimes residual stresses and deformations in the resulting product [14,15] make its further use simply unacceptable [16,17].

**Figure 1:**
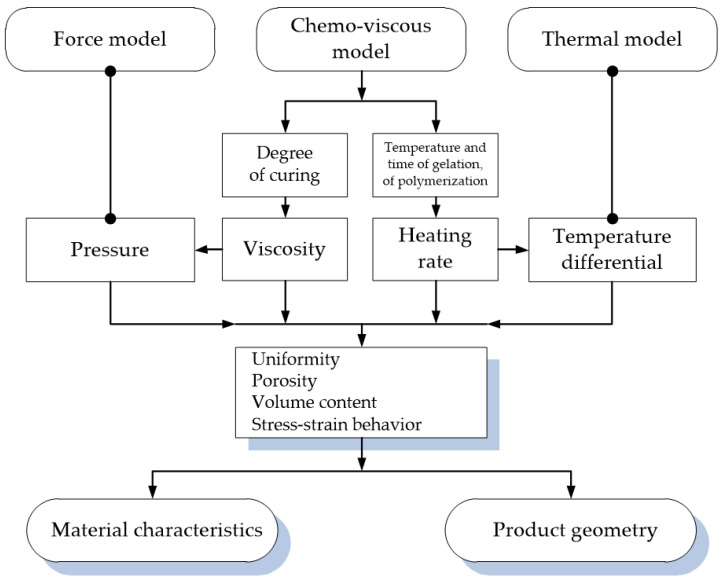
Interrelation of process parameters.

The process of moulding of PCM products cannot be completely modelled [18,19]. It is due to the complexity of the mathematical description of the processes that occur in the moulded PCM, as well as the necessity to consider their mutual influence (Figure 1). Therefore, the moulding of a composite product is usually considered with the use of several models describing the processes in the moulded material, such as chemo-viscous [20,21,22,23,24,25], thermal [26,27,28,29,30] and force [31,32,33,34,35,36] models. Mathematically, these processes are described independently of each other, and their mutual influence is considered as follows. The results obtained for one model represent the initial data to make calculations for the other one. For example, the link between the chemo-viscous model and the model of thermal action is the rate of temperature rise [25,28], while viscoelastic and viscous-flow characteristics at the time when pressure is applied and temperature change [19,29] represent the relation between the chemo-viscous model and the model of force action [37,38].

The chemical and physical processes that occur in the moulded material in the process of curing are described in [22,23]. The problems of chemical transformations in the binder and release of volatile products are solved, and the shrinkage processes are considered. Nevertheless, the impact of temperature on the characteristics of the material in the process of curing is not considered, and the influence of the dimensions and geometry of the product and moulding equipment is not analyzed. In recent years, studies related to the online monitoring of the process of impregnation of reinforcing material in the curing process have been conducted [20,21,39]. The results of the studies allowed for the simulating, controlling, and regulating of the curing process at the initial stage of moulding. However, the occurrence of the thermal stress-strain behaviour at subsequent stages was not considered. The phenomena of relaxation and creep that occurred in the composite at the stage of temperature holding and cooling were discussed in [24,27], but distribution of the thermal field in the process of curing was not studied in these papers. Temperature phenomena also participate in the occurrence of stresses and deformations at the stage of heating, along with shrinkage, according to [25]. It is shown that temperature stresses become comparable with shrinkage stresses upon reaching the material viscosity corresponding to 60 ... 70% conversion in the binder. The papers [28,29] describing the occurrence of stress-strain behaviour at the stage of PCM heating to the polymerization temperature based on experimental studies are of some interest. One of the primary issues in the modelling of the curing process is to determine the model for changing the degree of curing, and hence the viscosity in the composite at the stage of its transition from viscous flow to solid state [37,38]. However, these papers do not address the problems associated with the supply of additional heat to the composite, and they do not consider the influence of geometric parameters of the product and equipment. A particular case of the composite curing by the autoclave method is studied in [31]. In the process of the work with prepregs, the issues of optimal technological modes ensuring their regulated quality are solved [32]. In addition, the modes of technological processes are ambiguously harmonized with each other. Detailed consideration of these methods in the future allows us to project the results onto other methods or to generalize. The papers [12,33] deal with the nature, mechanism of occurrence and mode of technological defects, but the impacts of excessive pressure of the curing process and the processes of relaxation of stresses and creep are not considered. The technological modes of PKM moulding were experimentally investigated in [34]. The studies allowed for the increasing of the PCM strength by 25–35%. However, the results can be used only for a narrow class of fluoroplastic materials and quartz fabric. The process of moulding in [40,41] was modelled for the binder only; the influence of the reinforcing material was not considered, and the issues of cooling, rheology, and changes in the characteristics of PCM components that depend on the temperature were not addressed. The approach to the application of the principles of computer-aided design for choosing the optimal design-and-technological parameters in the moulding of PCM with the use of ultrasonic treatment has been developed in [42].

The moulding models proposed in [19,35,36] are of particular interest. The authors of these papers propose not only to solve the problem of choosing the rational process parameters of the moulding mode, but also to control pre-set parameters. The Newtonian fluid, flowing from a plane located between two rigid plates compressed by pressure and approaching at a certain speed, was taken as a model. However, the presented approach does not consider the binder flow into the absorbent layer. That is, the binder is not removed from the moulded material, but it only fills the voids in the PCM. In most cases, this nature of the flow leads to the uneven distribution of the binder in the moulded material, with the observed increase in the binder content in the edge zone [43,44].

The presented review shows several important theoretical and experimental studies, which were conducted to determine and reveal the patterns of physical and chemical processes during PCM product moulding. However, all these studies covered only the individual stages of the moulding process; the results were scattered, and stress-strain behaviour of the structure that occurs during its manufacture was not considered in many cases. Almost all the papers do not pay due attention to the determination of moulding pressure, although this parameter of the moulding process is one of the principal ones. Moulding pressure regulates the volume content of the reinforcing material and ensures the uniformity, degassing, and possible reduction in the residual stress-strain behaviour. The creation of a method for choosing the rational parameters of the moulding process (temperature, pressure, and time), materials of additional layers and equipment will allow us to predict the stress-strain behaviour of the composite structure at any time and at any point of the moulded material, even before the moulding begins. Moreover, the reduction in the cost of PCM products, which is an urgent task today, is to a certain extent associated with the minimization of high energy costs and time input inherent in moulding processes [19,45].

## 2. Materials and Methods

A study of the effect of pressure of the moulding process on the quality of a composite product was carried out based on the linear theory of thermoelasticity of anisotropic bodies. The technological choice of the level of moulding pressure was determined by the conditions for obtaining and maintaining the given structural parameters of PCM, which are as follows: regulated content of the reinforcing material in the structure, its solidity (absence of porosity in the material), as well as the given geometry. The solution to the force problem was reduced to determining the moment of time and magnitude of applied/released moulding pressure. At the first stage of the study, an analytical dependence was obtained to define the process parameters (pressure, time) providing a nonporous PCM structure. To reduce the PCM porosity, the process parameters (temperature, time and pressure) were selected in such a way that the maximum amount of volatile reaction products was released at the lowest viscosity of the curing binder. Furthermore, at the initial stage, with the lowest viscosity of the binder, the moulding pressure included the vacuum pressure only. We used the experimental data obtained earlier in [19,28,36] for the relative mass of the volatile-matter yield for prepregs based on phenol-formaldehyde binder BFOS, epoxy-phenol binder 5-211-B, epoxy binder ENFB (producer: Federal State Unitary Enterprise All-Russian Scientific Research Institute of Aviation Material, Russian Federation) and phenol binder LBS-4 (producer: TD Holding Company “FEM”, Russian Federation) depending on the temperature and time of holding at this temperature. The resulting dependence was used to determine the temperature and time of the degassing process start and duration. At the next stage, the analytical dependence for the determination of the required pressure from the condition of achieving the regulated PCM volume content, and pre-set geometric parameters of the composite product, was obtained. The moment of time when the binder viscosity was close to minimum, and the yield of the volatile fractions already exceeded the maximum value was considered the initiation of the application of the pressure. The model fluid filtration in a porous medium, which is described by Darcy’s law in a one-dimensional setting [19,36], was adopted as a model, describing the process of binder flow in the PCM package. It was assumed that permeability of the medium was a function of the porosity, volume content of the reinforcing material (in case of laminated material), fibre diameter, angle of reinforcement and type of weaving of the material. The presented dependence allowed us to unambiguously determine the dependence of the moulding pressure on the binder viscosity and the temperature at which the pressure was applied, the structure of both the moulded PCM package and absorbent layer. At the cooling stage, the required pressure was determined from the conditions that would compensate for the stresses arising in the material at the stage of cooling, and thereby ensure their relaxation without an increase in deformations (creep). The value of the required pressure was determined according to the calculation model of a rod on the elastic foundation and the known dependencies obtained in [36]. Because it is rather difficult to implement a variable pressure in the plane from the technological point of view, the value of the moulding pressure was assumed to be constant. It corresponded to the maximum deflection of the moulded composite panel. Experimental studies were carried out in the laboratory conditions using the standard equipment, instruments, and fixtures (thermostat TERMOTET 04/2, SNOL 60/300 NL curing oven, computer with the special soft). The kinetics of the PCM moulding process was studied based on the experimental data obtained by the standard methods of chemical and electro-physical analysis. The experimental studies of the influence of several factors (rates of heating and cooling, moulding pressure, and time) on the residual deflection of the PCM panel were carried out on specimens of plates of 150 × 150 mm. The specimens were made of prepreg based on T-10-14 glass cloth (producer: JSC “Polotsk-Steklovolokno”, Republic of Belarus) on an FP-520 (producer: Federal State Unitary Enterprise All-Russian Scientific Research Institute of Aviation Material, Russian Federation) binder by manual layup onto the flat mould. The relative volume content of the binder in the prepreg was 53 ± 5%. The moulded PCM package was of symmetrical structure (0°; 90°; 90°; 0°). Deflections were measured by photographs. The specimen was fixed before the lens in the way that the curved plate located in front of the lens was projected into a clear curve. Theoretical and experimental studies allowed us to develop a method for determining the rational parameters (pressure, time) of the moulding process.

## 3. Theoretical Background

The technological choice of the level of moulding pressure is determined by the conditions for obtaining and maintaining the given structural parameters of PCM, which are as follows: regulated content of the reinforcing material in the structure, its solidity (absence of porosity in the material), as well as the given geometry [18,19].

To ensure the geometry of the structure and regulated content of the reinforcing material in it, it is necessary to deform the workpiece accordingly. Irreversibility of the deformation is provided by removal of excess binder from the workpiece into absorbent layers of auxiliary equipment, or by uniform spreading of the binder and filling of vthe oids. After the removal of excess binder and compaction of the material, the deformed state of the workpiece should correspond to the product shape. In this case, a solution to the force problem is reduced to determining the moment of time and magnitude of applied/released moulding pressure. This solution is closely related to solving chemo-viscous and thermal problems, since the applied pressure will guarantee the specified volume content of the reinforcing material, geometry, and solidity of the product. The moment of application of the moulding pressure is associated with the change in viscosity of the material and removal of volatile products; therefore, it can be determined only after solving the chemo-viscous problem.

One of the main defects of the finished product is the non-uniform structure of the matrix [46]. Non-uniformity is often caused by the incomplete removal of the volatile fractions contained in solvents or partial removal of polycondensation products. The structure of the volatiles are solvents in the gaseous phase, and in some cases, they are also low molecular weight polycondensation products, such as water molecules. The low molecular weight fraction, as well as the solvent, are removed as a rule at the stage of vacuum [30]. The volatile products begin to form intensively after the gelation point [28,45], and the conditions leading to the formation of a porous structure may arise. In the first case, the porosity of the material may occur at the vacuumizing stage. Here, the viscosity of the binder is low, and the pressure of the volatile products dissolved in the curing polymer will be less than the external pressure. Removal of the volatile fractions will be prevented by the external pressure, causing the formation of a porous structure in the moulded material, which worsens its physic-mechanical properties. In the second case, pores in the moulded material may appear when the binder reaches high viscosity at the pressure of the volatile fractions exceeding the external one. This situation is typical for processes with the rapid change in viscosity. In this case, due to the high viscosity of the binder and high pressure of the volatile fractions, the material boils up with the formation of a spongy structure.

Therefore, to reduce the PCM porosity, it is necessary to choose the process parameters (temperature, time, pressure) in such a way that the maximum amount of volatile reaction products is released at the lowest viscosity of the curing binder. At the initial stage, with the lowest viscosity of the binder, the moulding pressure should include the vacuum pressure only. This vacuum pressure helps to remove volatile fractions from the binder. An example of the dependence of volatile matter yield on the rate of temperature rise in the process of curing is given in (Table 1) [19,28,36].

The holding time depends on the mass of volatile fractions in the moulded PCM package and the rate of their release. The holding time is defined as follows:

(1) $$\tau_{v}=\frac{m_{v}}{v_{v}},$$

where *m_v_* is the mass of the volatile-matter yield; *v_v_* is the rate of release of the volatile matter.

**Figure 2:**
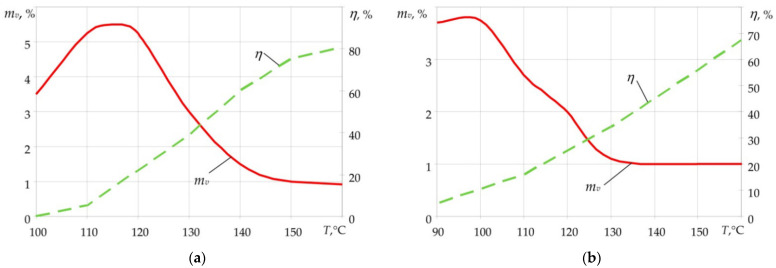
Dependence of volatile-matter yield (*m_v_*) and degree of curing (*η*) on the heating temperature for LBS-4 (**a**) and ENFB binders (**b**).

Figure 2 [19,28,36] shows the data on the relative mass of the volatile-matter yield for prepregs based on LBS-4 and ENFB binders depending on the temperature and time of holding at this temperature. From the presented data, we can observe the following. The peak of the maximum volatile-matter yield for these binders lies in the temperature range in which the degree of curing does not exceed 10%. Consequently, the viscosity value remains virtually unchanged, which means it will not prevent the release of volatiles from the binder.

At lower temperatures, the reaction rate is low; therefore, fewer volatile products are released. At higher temperatures, with the reaction rate increase, the viscosity of the substance also increases; as a result, volatile matter remains inside the binder, forming the porous structure of the material [8,10].

To ensure the geometry of the structure and regulated content of the reinforcing material therein, it is necessary to deform the workpiece accordingly. Irreversibility of the deformation is provided by the removal of excess binder from the workpiece into the absorbent layers of the auxiliary equipment, or by uniform spreading of the binder and filling of the voids. After the removal of excess binder and compaction of the material, the deformed state of the workpiece should correspond to the product shape. At the same time, it is important to create the conditions in the moulded material (product) where the liquid will only move along the normal to the outer/inner contour, until the pressure in the absorbent layer and the moulded PCM package is equalized. Otherwise, movement of the binder over the volume of the moulded PCM package begins, causing its uneven distribution [47].

**Figure 3:**
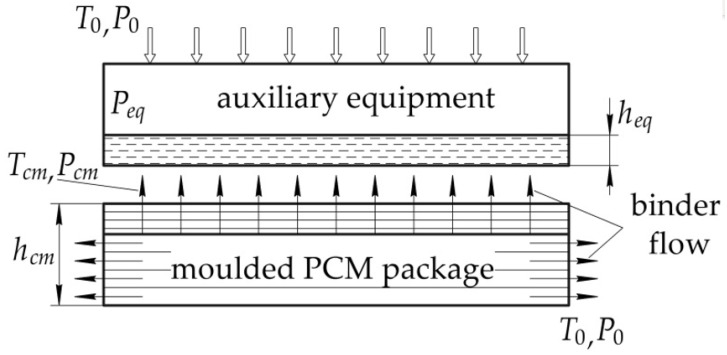
Diagram of moulding pressure application.

We consider the beginning of the pressure application to be the time when the binder viscosity is close to the minimum, and the yield of the volatile fractions already exceeds the maximum value. The model fluid filtration in a porous medium, which is described by Darcy’s law in a one-dimensional setting (Figure 3) [19,36], is adopted as a model, describing the process of binder flow in the PCM package.

The expression for the moulding pressure is written as

(2) $$q=-\frac{K}{\mu }\frac{dP}{dx},$$

where *K* is the permeability of the medium; *μ* is the viscosity.

Permeability of the medium is a function of porosity, volume content of the reinforcing material (in case of laminated material), fibre diameter, angle of reinforcement and type of material weaving. It is defined by the following relationship:

(3) $$K=-\frac{d^{2}}{k_{0}}\frac{{\left(1-\Theta_{r}\right)}^{3}}{\Theta_{r}^{2}},$$

where *d* is the fibre diameter; *k*_0_ is the empirical coefficient considering the reinforcing material structure; Θ*_r_* is the volume content of the reinforcing material.

Coefficient *k*_0_ will be different for each type of weaving and reinforcing angle. For a unidirectional material in the longitudinal direction, *k*_0_ is equal to 0.5…0.7, for the same material in the transverse direction *k*_0_ = 11; for the woven material and mat, *k*_0_ takes the value of 5.5 [36].

The viscosity of the binder depends on the degree of curing. At the initial stage of heating of the material, when the binder softens, viscosity of the material decreases. At the gelation point, a liquid stops flowing due to crosslinking and the viscosity approaches infinity. However, after reaching the gelation temperature, the viscosity value begins to increase.

Equation (2) is rewritten in the integral form as the following two equalities:

- Pressure in the absorbent layer
  (4) $$q_{eq}=\frac{K_{eq}}{\mu \left(\eta ,t\right)}\frac{\left(p_{0}-p_{eq}\right)}{h_{eq}},$$
- Pressure in PCM
  (5) $$q_{cm}=\frac{K_{cm}}{\mu \left(\eta ,t\right)}\frac{\left(p_{eq}-p_{cm}\right)}{h_{cm}},$$
  where *K_cm_*, *K_eq_* are the permeability of the moulded PCM package and auxiliary equipment, respectively; *h_cm_* is the thickness of the layers of the PCM package with the regulated content of the binder; *h_eq_* is the thickness of the auxiliary equipment with the binder; *p*_0_, *p_cm_*, *p_eq_* are the applied pressure, pressure in the PCM layers and pressure in the auxiliary equipment, respectively.

Change in the PCM volume is defined from the continuity equation, which is as follows:

(6) $$-\frac{d\left(hS\right)}{dt}=Sq_{eq}=Sq_{cm},$$

where *S* is the PCM package surface area; *h* is the total thickness of the PCM package.

Pressure is related to the applied load by the following relationship:

(7) $$p_{0}=\frac{F}{S}+p_{a},$$

where *F* is the applied force; *p_a_* is the atmospheric pressure.

After substitution of expressions Equations (6) and (7) into Equation (5), we obtain the dependence of the change in the PCM volume over time, which is as follows:

(8) $$-\frac{d\left(hS\right)}{dt}=\frac{K_{eq}K_{cm}F}{\mu \left(\eta ,t\right)\left(K_{eq}h_{cm}+K_{cm}h_{eq}\right)}$$

With the area of the moulded composite product being constant, the required pressure is defined as follows:

(9) $$p_{0}=\frac{\mu \left(\eta ,t\right)\left(K_{eq}h_{cm}+K_{cm}h_{eq}\right)}{K_{eq}K_{cm}}\frac{dh}{dt}-p_{a}.$$

Using the dependence (9), we can determine the time of application of the pressure until a structure with the given volume content is obtained. In addition, the presented relation unambiguously determines the dependence of the moulding pressure on the viscosity of the binder and the temperature at which pressure is applied, as well as the structure of both the moulded PCM package and the absorbent layer.

At the stage of cooling of the composite construction, when the process of formation of the PCM structure is almost complete, it is necessary to fix the obtained characteristics. Characteristics at the stage under consideration can be fixed with the use of process parameters, such as the rate of temperature change, stepwise cooling of the product and the required pressure. Fixation of the obtained product characteristics by the cooling rate will lead to a delay in the process and additional costs, and implementation of stepwise cooling, in some cases, can aggravate the situation because of the material creep [19,20,36]. Therefore, it is advisable to carry out the cooling stage at a certain required pressure to compensate for the emerging stresses in the material at the cooling stage, and thereby ensure their relaxation without an increase in deformations (creep).

**Figure 4:**
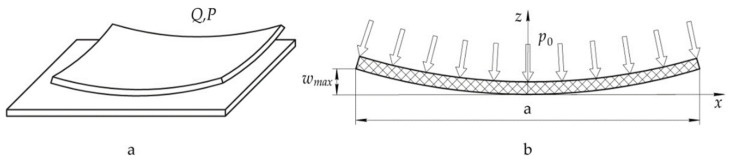
Diagram of pressure application at the cooling stage (**a**) and adopted calculation model of a rod on the elastic foundation (**b**).

The value of the required pressure is determined by a well-known method and dependencies (Figure 4) [36,48].

In this case, the calculated dependence of the required pressure is as follows:

(10) $$p_{0}=-w_{max}\beta^{3}D\cdot ch\left(\beta x\right)cos\left(\beta x\right),$$

where *w_max_* is the maximum deflection of the panel; *D* is the cylindrical rigidity of the panel in the given direction; *k* is the coefficient of stiffness of the base; $\beta =\sqrt[{4}]{\frac{k}{4{\left(EI\right)}_{cm}}}$; ${\left(EI\right)}_{cm}$ is the bending stiffness of the panel in the given direction.

Since it is rather difficult to implement a variable pressure in the plane from the technological point of view, the value of the pressure is assumed to be constant. Furthermore, it will correspond to the maximum deflection of the panel. In this case, the value of the required pressure may be somewhat overestimated in some areas, possibly causing the deformation of fibres of the reinforcing material and change in specified characteristics of the product. In addition, it may also lead to additional energy costs.

## 4. Experimental Research

The effect of moulding modes on the residual deflection of the panel of PCM was studied experimentally. Specimens of plates of 150 × 150 mm were made of prepreg based on T-10-14 glass cloth on a FP-520 binder by manual layup onto the flat mould. The moulded PCM package is of symmetrical structure (0°; 90°; 90°; 0°). Physic-mechanical characteristics of the material were as follows: elastic modulus in the longitudinal direction *E*_1_ = 30.8 GPa; elastic modulus in the transverse direction *E*_2_ = 25.5 GPa; shear modulus in the plane of the material *G*_12_ = 3.1 GPa; Poisson’s ratio *μ*_12_ = 0.28; density *ρ* = 1700 kg/m^3^; coefficient of linear thermal expansion in the longitudinal direction *α*_1_ = 4 · 10^−6^ K^−1^; coefficient of linear thermal expansion in the transverse direction *α*_2_ = 6 · 10^−6^ K^−1^; specific heat capacity c = 850 J/(kg·K); thermal conductivity coefficient λ = 0.3 W/(m·K); monolayer thickness 0.21 mm.

**Figure 5:**
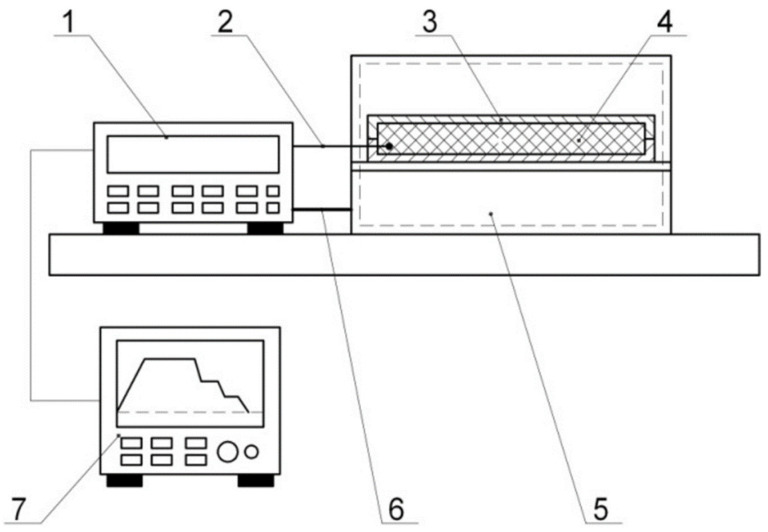
Test bench layout: 1—thermostat (TERMOTET 04/2); 2—thermocouple; 3—shaping surface; 4—moulded PCM package; 5—SNOL 60/300 NL curing oven; 6—supply; 7—data recorder (computer with the special soft).

Shaping was performed on the flat fixture that represented a polished steel plate of 10 mm thick. The degreased surface of the fixture was coated with an antiadhesive layer of K-21 lubricant, on which the layers of the moulded PCM package were successively laid according to the reinforcement pattern. The layers arranged as above were pressed with a roller; vacuum cover fixed along the perimeter with a putty yarn was placed on the laid PCM package. The prepared package was vacuumized and put into the heater (Figure 5).

Upon completion of the moulding process, an external examination of the specimens was carried out with the recording of visible defects. After that, the specimen deflection was measured using the obtained photographs. The specimen was fixed before the lens in the way that the curved plate located in front of the lens was projected into a clear curve.

**Figure 6:**
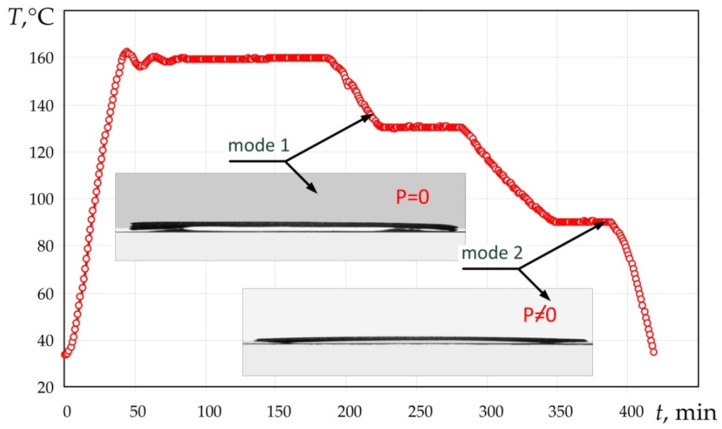
Moulding modes No 1 and No 2 and glass-fibre reinforced plastic specimens obtained by these modes.

The specimen of the glass-fibre reinforced plastic panel produced by the moulding mode No. 1 was moulded as shown on the graph (Figure 6).

The heating rate was 3.7 °C/min; the specimen was cooled in the following two stages:

- Cooling to the temperature of 130 °C at the rate of 1 °C/min.
- Isothermal holding at the temperature of 130 °C for 60 min.
- Cooling to the temperature of 90 °C at the rate of 0.7 °C/min.
- Isothermal holding at the temperature of 90 °C for 45 min.
- Cooling to the final temperature at the rate of 3.7 °C/min.

It should be noted that there was no vacuum pressure at the cooling stage.

The specimen obtained by moulding mode No 2 was moulded according to mode No 1; however, the vacuum pressure was removed only after the completion of the moulding process (Figure 6).

**Figure 7:**
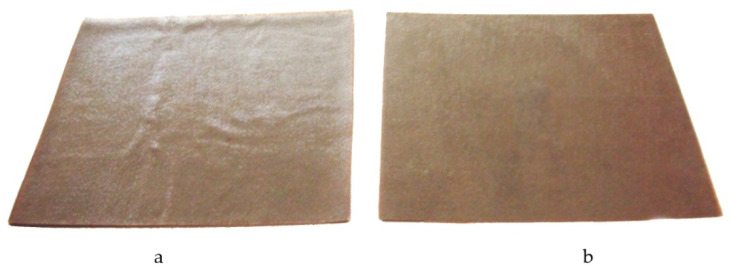
Glass-fibre reinforced plastic specimens obtained by moulding modes No 1 (**a**) and No 2 (**b**).

The external comparison of the specimens moulded according to the considered modes is shown in Figure 7.

The results of the experiment are presented in Table 2.

The specimen obtained during the moulding mode (Figure 7a) had several visible defects, namely the severe delamination of edges, poor adhesion of upper layers, surface with waviness and swelling areas. In the transverse direction, an area with the noticeable delamination of the upper layer from the adjacent one passed through the central part. The specimen deflection was equal to 3 mm. In contrast to the specimen obtained by moulding mode No 1, no defects were observed on the surface of specimen obtained by moulding mode No 2 (Figure 7b). The specimen obtained with the use of the moulding mode had a smooth surface, dense structure, and no visible defects (delamination of edges, waviness, swelling) were observed; deflection of the resulting specimen was 1.8 mm.

## 5. Results and Discussion

**Figure 8:**
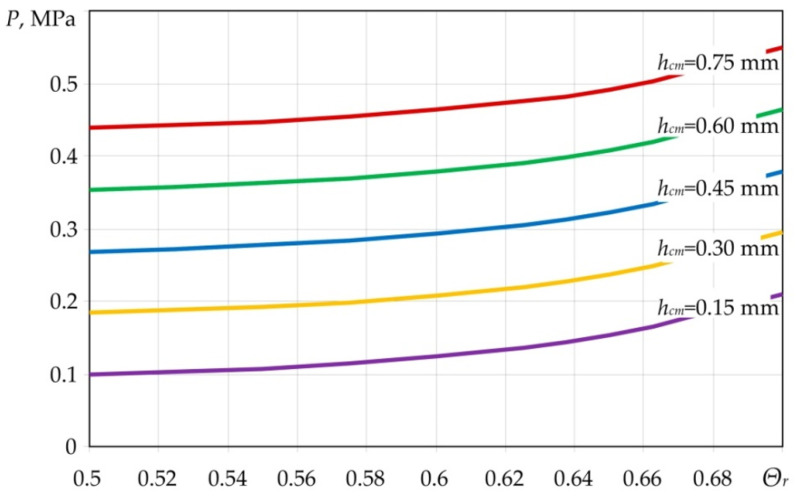
Dependence of the required moulding pressure on volume content of the reinforcing material in the product for different thicknesses of the PCM package layers.

We considered the moulded composite panel with the surface area of 100 × 100 mm, with T-10-80 glass cloth used as the product material, LBS-4 binder, specified thickness of the moulded package 1.5 mm, monolayer thickness 0.15 mm, structure (±45°), volume content of the reinforcing material in the finished product Θ*_r_* = 0.7, initial thickness of the moulded PCM package 1.7 mm, pressure supply temperature 100 °C, and viscosity of the binder of 6.7 Pa·s at the temperature of 100 °C. After analysis of the dependence of Equation (9), we can say that with the increase in the binder viscosity, the value of the required moulding pressure also increases. Moreover, it is the linear dependence as can be observed from Equation (9). With the increase in the value of the volume content and thickness of the moulded product (Figure 8), the value of the required moulding pressure also increases.

This is because the reinforcing material prevents free flowing of the binder over the volume of the moulded PCM package, as well as into the absorbent layer. It should be noted that thickness of the absorbent layer *h_eq_* cannot be less than the specified value. At the same time, it depends on the initial and final volume content of the reinforcing material in the PCM and the moulding pressure, and can be determined at the stage of development of the moulding as the following equation:

(11) $$h_{eq}=\frac{K_{eq}}{K_{cm}}\frac{\left[\left(p_{0}+p_{a}\right)\tau K_{cm}-\mu \left(\eta ,t\right)h_{cm}^{2}\right]}{\mu \left(\eta ,t\right)h_{cm}}.$$

The thickness of the absorbent layer being less than the value obtained according to Equation (11) leads to the saturation of the absorbent layer. After that, the binder begins to redistribute over the volume of the moulded material, which can result in the formation of discrete areas with high contents of the binder [9,46].

Consequently, to determine the required moulding pressure during heating, we can use the developed model Equation (9) as the model describing the conditions for application of pressure, depending on the geometric parameters of the materials included in the composition, as well as the changes in the viscosity of the binder during moulding. In addition, dependence Equation (9) allows us to choose the geometric parameters of the absorbent layer, as well as its structure.

After the comparison of the results obtained experimentally and theoretically, we can conclude the following. The error between the theoretical values of deflections obtained at the temperature of 20 °C $w_{t}^{20\;{}^{\circ}C}$ and experimental values of deflections (*w_e_*) for the specimen obtained by the mode No 2 is overestimated by 36% (Table 3). For the specimen obtained by the mode No 1, the deflection value turned out to be underestimated by 6.5%. Overestimation of the theoretical value of deflection is primarily due to neglecting the temperature dependence of the PCM physic-mechanical characteristics, as well as the influence of creep and relaxation.

In case when the deflections were determined considering the change in the PCM physic-mechanical characteristics that depend on the temperature $w_{t}^{160\;{}^{\circ}C}$ [36,45], error values of the theoretical and experimental results for specimens No 1 and No 2 were 16.7 and 30%, respectively. A high error value in this case can be explained by stepwise cooling during the experiment; it allowed for the realizing of the unrecorded rheonomic properties of the material.

Determination of the theoretical values of deflections for the specimens was also performed based on the change in characteristics, considering the rheonomic properties [36,45]. For the specimen moulded by the mode No 1, the deflection was determined taking the creep into account, and for that moulded by the mode No 2, the relaxation according to known dependences [36,49] was considered. In this case, errors of the theoretical values of deflections ($w_{t}^{R}$) and experimental values for the specimens moulded by the modes No 1 and No 2 were 2.7% and 4.2%, respectively.

**Figure 9:**
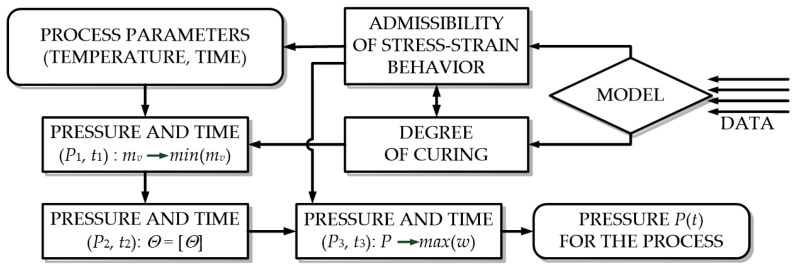
Schematic diagram of the method for the determination of the rational process parameters (pressure, time) of the moulding process.

Theoretical and experimental studies allowed us to develop a method for the determination of the rational process parameters (pressure, time) of the moulding process (Figure 9).

At the initial stage, the input parameters are set, such as PCM characteristics (physic-mechanical characteristics, structure, and thickness), characteristics of the equipment and range of possible moulding pressure values. Then, the following steps are carried out:

1. Following the technique described in [28,45], the degree of binder curing in the temperature-time range of the moulding process and viscosity depending on the degree of curing of the material are determined.
2. The time and pressure are calculated from the condition of the maximum vola-tile-matter yield of the binder. The degassing time of the PCM package is determined by the formula (Equation (1)).
3. The value of the required moulding pressure is determined depending on the characteristics of the auxiliary layers, moulded material and reaching of a certain degree of volume content of the reinforcing material. The required moulding pressure is determined from the condition of obtaining a structure with the given volume content of the components according to formula (9).
4. The required moulding pressure is determined from the condition of the structure fixation (Equation (10)).
5. If the difference in pressure values obtained in paragraphs 2–4 does not exceed 10%, then the required pressure is chosen as the maximum of those obtained.
6. If the obtained pressure values in paragraphs 2–4 differ significantly from each other, the specific value is taken for each section.
7. The time of the moulding pressure release will be the time of the moulding process completion.

**Figure 10:**
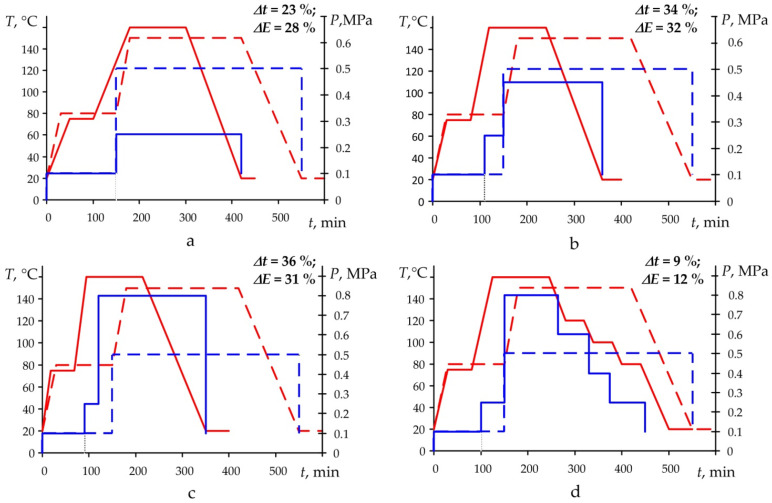
Mode of moulding of glass-fibre reinforced plastic panels (150 × 150 mm) at different heating and cooling rates: (**a**) 1 °C/min; (**b**) 2 °C/min; (**c**) 3 °C/min; (**d**) stepwise cooling, ––– proposed moulding temperature; – – – standard moulding temperature; ––– proposed moulding pressure; – – – – standard moulding pressure; ∆*t*, ∆*E*—time and energy saving compared to the standard moulding mode.

The results of the implementation of the developed method are shown in Figure 10.

Comparing the results, we can conclude the following. At a 1 °C/min rate of change in the temperature of the PCM and the panel, the pressure value of the entire moulding process can be taken constant, being equal to 0.25 MPa (Figure 10a). In this case, the moulding time is reduced by 23% and consumed energy by 28%. If the rate of temperature change during the heating and cooling stages is 2 °C/min, the moulding pressure should first be raised to 0.25 MPa, and after the degassing stage, it is increased to 0.5 MPa (Figure 10b), and 34% of time and 32% of energy can be saved. For the mode of panel moulding at the temperature change of 3 °C/min, the pressure supply mode is the same as the mode implemented at the rate of 2 °C/min, but the required pressure after degassing should be at least 0.8 MPa (Figure 10c). Here, the time of moulding will increase by 2%, and consumed energy will decrease by 1%. At the stage of cooling of the composite structure, when the process of PCM structure moulding is almost completed, stepwise cooling of the product and stepwise pressure reduction were implemented to fix the obtained characteristics (Figure 10d). It allowed us to provide the additional relaxation of the PCM panel without an increase in its deformations. The time of moulding compared to the standard moulding mode is reduced by 9%, and consumed energy by 12%.

## 6. Conclusions and Further Research

In this research, the following results were obtained:

The analytical dependence has been obtained, which allowed us to determine the process parameters (pressure, time), which ensure the nonporous structure of PCM. This dependence is used to determine the temperature and time of the degassing process start and duration.

In addition, the analytical dependence has been obtained for determining the re-quired pressure from the condition of achieving the regulated volume content of PCM, as well as the specified geometric parameters of the composite product. The proposed dependence considers the mutual influence of the parameters of the auxiliary equipment and the moulded material and its structure (layup angle and type of weaving).

The approach is proposed to determine the parameters of the absorbent layer from the condition of reaching the given volume content in the PCM package.

The method has been developed to determine the rational process parameters (pressure, time), which ensure the achievement of the uniform nonporous structure of the given geometry. A clear relationship has been established between the parameters of the moulding process (pressure, time) and characteristics of the moulded material, depending on the temperature and time.

The experimental results indicate that the moulding process should be carried out in the presence of pressure. The experiments proved the presence of several defects, such as the delamination of edges, waviness, swelling and poor adhesion of upper layers in the specimen of the composite panel cooled stepwise in the absence of the vacuum pressure. The surface quality of the specimen of the panel cooled stepwise under vacuum pressure was significantly better, and no visible defects were observed. The obtained theoretical values of deflections, considering the change in physic-mechanical characteristics depending on the temperature and rheonomic properties of the material, showed an error that did not exceed 7%, compared to the experimental data.

The results of the work can be applied at the enterprises engaged in designing and manufacturing panel structures of PCM.

## Figures and Tables

**Table 1:** Dependence of volatile-matter yield (%) on the rate of temperature rise in the process of curing.

Rate, °C/min	Binder			
	BFOS	5-211-B	ENFB	LBS-4
0.5	76.4	42.00	39.78	44.15
1.0	76.2	41.55	40.16	43.15
1.5	76.1	40.25	39.70	43.10
2.0	76.06	39.85	39.78	42.60
2.5	74.88	40.37	39.30	41.07
3.0	73.28	37.50	39.00	38.08

**Table 2:** Processing of results of the experiment.

Moulding Mode	Heating Rate, °C/min	Cooling Rate, °C/min	Deflection, mm	Notes
No 1	3.7	1; 0.7; 3.7	3	There is no vacuum pressure at the cooling stage. Surface is wavy with swellings. Significant delamination of edges and delamination of the upper layer is observed.
No 2	3.7	1; 0.7; 3.7	1.8	Surface is smooth, without any visible defects.

**Table 3:** Theoretical and experimental data processing.

**Moulding Mode**	$w_{e},$ mm	$w_{t}^{20\;{}^{\circ}C},$ mm	$w_{t}^{160\;{}^{\circ}C},$ mm	$w_{t}^{R},$ mm	$\frac{w_{t}^{20\;{}^{\circ}C}-w_{e}}{w_{e}}100\%$	$\frac{w_{t}^{160\;{}^{\circ}C}-w_{e}}{w_{e}}100\%$	$\frac{w_{t}^{R}-w_{e}}{w_{e}}100\%$
1	3	2.82	2.57	3.08	6.4	16.7	2.7
2	1.8	2.82	2.57	1.72	36.2	30.0	4.2

## Data Availability

Not applicable.
